# Editorial Correspondence

**Published:** 1878-01

**Authors:** R. W. Westmoreland

**Affiliations:** Fulton, Ark.


### EDITORIAL CORRESPONDENCE.

---

Fulton, Ark., Jan. 17, 1878.

*Editor Atlanta Medical and Surgical Journal:*

I have noticed with a great deal of interest an essay on belladonna, with theoretical remarks as to its *modus operandi* published, in the last issue of the Journal. There are in it many ideas that are valuable in my estimation, and I shall avail myself of the first opportunity of testing the efficacy of the drug in some of the affections for which the essayist advises its administration. Of its virtues as an antihydrotic, I would have been made very glad had I known of it in a case o "sweaty" hands that you know of. The subject has despaired of ever being cured, and I would be glad to learn that you had tested the power of belladonna in relieving him. I can say unhesitatingly that in incontinence of urine from an irritable os cystis (?) from whatever cause, belladonna, in my experience, acts "like a charm." Then, too, in spasmodic affections, resulting from centric or peripheral irritation, or as a result of loss of tone from some constitutional drain or other source of debility, I have found belladonna to act very beneficially. The physiological action, I would be inclined, from these facts, to suppose, consisted in its stimulant effects upon the cerebrum. (The distinct action it may exert upon the ganglionic system may give us a clue as to its potency in certain glandular affections, while its topical administration may have its advantages through the influence wrought upon the inhibitory and trophic branches supplying the part.)

As an item of curiosity, I will give you the description of an eyeless cat I have just seen—now two years old. It is perfectly eyeless—has never had any eyes, though the orbits and lids are perfectly formed. The orbital cavity is regular and of normal dimension, apparently, and the tissues in healthy condition. I inquired particularly into the history, and elicited the information that it had been under sufficient attention since its birth to warrant the assertion that *its eyes* had never been injured or diseased; at least there had been no indication of such a condition. To see the thing, one would scarcely think that it was sightless. It seems to get about as well as any cat, and turn a crippled bird loose in the yard, it can catch it, apparently, as quick as a cat *ought to.*

We are having a great deal of pneumonia now, presenting pretty much always the asthenic type, and generally fatal. My plan is to give stimulants and opiates with belladonna, combined with some counter irritation, in the first stage. Occasionally, when the fever runs high, veratrum is given in two to three-drop doses. On account of the tendency to congestion as a result of debility, I have thought of administering ergot. What do you think of the idea? Upon the theory of its beneficial effect upon the muscular coats of the vessels, I would think such a course reasonable.

Rheumatism in all its varied forms abounds here. I have had, as yet, no occasion to abandon the general treatment of quinine and counter irritation. Catalytics more potent than quinine are chemically indicated often to correct the blood dyscrasia; but for the nervous condition, I rely upon irritation and cups over the spinal column.

Respectfully, R. W. Westmoreland, M.D.

